# (Patho-)Physiology of Na^+^/H^+^ Exchangers (NHEs) in the Digestive System

**DOI:** 10.3389/fphys.2019.01566

**Published:** 2020-01-15

**Authors:** Li Cao, Zhenglin Yuan, Mei Liu, Christian Stock

**Affiliations:** ^1^Department of Gastroenterology, Tongji Hospital, Tongji Medical College, Huazhong University of Science and Technology, Wuhan, China; ^2^Department of Stomatology, Union Hospital, Tongji Medical College, Huazhong University of Science and Technology, Wuhan, China; ^3^Department of Gastroenterology, Hepatology and Endocrinology, Hannover Medical School, Hanover, Germany

**Keywords:** Na^+^/H^+^ exchangers, physiology, malfunction, pathophysiology, digestive system

## Abstract

Na^+^/H^+^ exchangers (NHEs) are expressed in virtually all human tissues and organs. Two major tasks of those NHE isoforms that are located in plasma membranes are cell volume control by Na^+^-uptake and cellular pH regulation by H^+^-extrusion. Several NHEs, particularly NHE 1–4 and 8, are involved in the pathogenesis of diseases of the digestive system such as inflammatory bowel disease (ulcerative colitis, Crohn’s disease) and gastric and colorectal tumorigenesis. In the present review, we describe the physiological purposes, possible malfunctions and pathophysiological effects of the different NHE isoforms along the alimentary canal from esophagus to colon, including pancreas, liver and gallbladder. Particular attention is paid to the functions of NHEs in injury repair and to the role of NHE1 in Barrett’s esophagus. The impact of NHEs on gut microbiota and intestinal mucosal integrity is also dealt with. As the hitherto existing findings are not always consistent, sometimes even controversial, they are compared and critically discussed.

## Introduction

The human Na^+^/H^+^-exchanger (NHE; SLC9) family comprises 13 proteins: SLC9A1-SLC9A9 (NHE1-9), SLC9B1 and B2 (NHA1, NHA2), and SLC9C1 and C2 (Fuster and Alexander, 2014). Occasionally, SLC9C1 and C2 are also called NHE10 and NHE11, respectively (Fuster and Alexander, 2014). NHEs are differentially expressed in virtually all human tissues and organs that are characterized by their specific physiological functions (Mine et al., 2015; Xu et al., 2018).

NHE1, for example, has been located in the basolateral membrane of polarized epithelial cells, where it is responsible for intracellular pH homeostasis, Na^+^ uptake and the maintenance of cell volume (Cetin et al., 2004). On the other hand, NHE1 was found to accumulate in the leading edge of migrating cells (Grinstein et al., 1993; Shi et al., 2013), particularly in invadopodia of malignant tumor cells of epithelial origin. NHE1 contributes to cell motility, both structurally as a cytoskeletal anchor and functionally by regulating pH and cell volume. Especially intra- and extracellular pH nanodomains in close proximity to the plasma membrane affect cytoskeletal dynamics, cell adhesion and the activity of MMPs (Stock and Pedersen, 2017).

**FIGURE 1:**
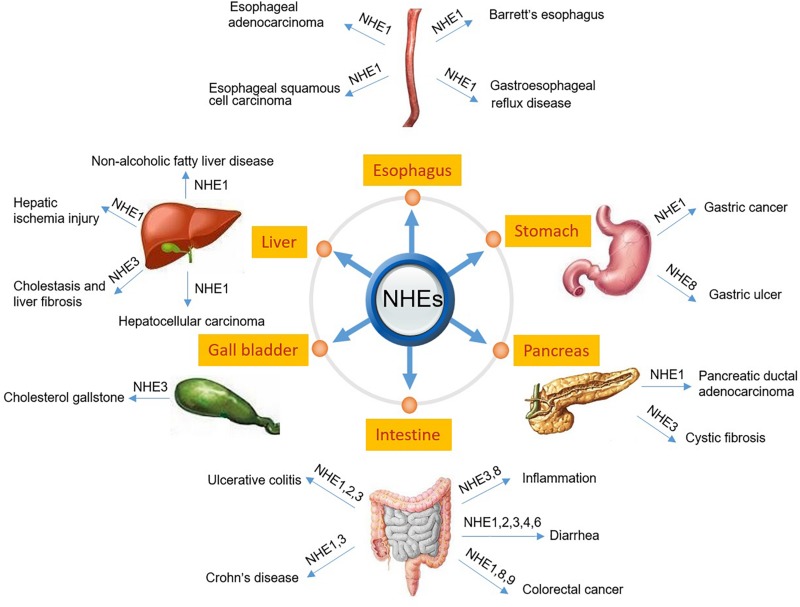
Schematic diagram depicting NHEs that have been known to be related to the pathogenesis of digestive diseases.

The digestive system consists of the gastrointestinal tract including esophagus, stomach, small intestine and large intestine, as well as the auxiliary organs: tongue, salivary glands, pancreas, liver and gallbladder. Several NHE isoforms are involved in the pathogenesis of diseases of the digestive system such as IBD (Magalhaes et al., 2016) or digestive system tumorigenesis (Kong et al., 2014). However, while the vast majority of review articles focuses on the role of NHE family members in IBD (Magalhaes et al., 2016) or infectious diarrhea in gut (Das et al., 2018), so far, only a few reviews report on the role of NHEs in digestive system tumorigenesis. Therefore, in this review, we will describe the pathophysiology of NHEs in the digestive system, including injury repair, infectious diarrhea, IBD, digestive system tumorigenesis, and others. Table 1 and Figure 1 summarize the information given throughout the text and include the relevant references.

**TABLE 1:** NHE isoforms, their locations, (patho)physiological impact, and relevant references.

**NHE isoforms**	**Digestive organ**	**Physiological purpose/Expression/(Mal)Function**	**Pathological manifestation**	**References**
NHE1	Esophagus	• To protect esophageal cells from the detrimental effects of gastric acid • Basolateral acidification of extracellular space and tissue damage • Increased NHE1 expression/activity defends intracellular pH • Na^+^-dependent Ca^2+^-extrusion • Proliferation, migration, invasion, and apoptosis	• GERD • GERD • Barrett’s esophagus • Esophageal adenocarcinoma • ESCC	Shallat et al., 1995; Park et al., 2015 Shallat et al., 1995; Siddique and Khan, 2003 Fitzgerald et al., 1998; Ostrowski et al., 2007; Goldman et al., 2010; Laczko et al., 2016 Goldman et al., 2011; Guan et al., 2014 Ariyoshi et al., 2017
NHE1	Stomach	• Initiation of cell migration during gastric epithelial restitution • Not essential for mouse gastric epithelial repair • Insulin-like growth factor II-induced proliferation and carbachol-/insulin-like growth factor II-stimulated migration in human gastric myofibroblasts • Proliferation, migration, and invasion of gastric cancer cells	• Gastric epithelial repair • Gastric epithelial repair • Gastric epithelial repair • Gastric cancer	Paehler Vor der Nolte et al., 2017 Xue et al., 2011 Czepán et al., 2012 Liu et al., 2008; He et al., 2010; Nagata et al., 2011; Hosogi et al., 2012; Xia et al., 2016; Xie et al., 2017
NHE1	Intestine	• NHE1-mediated Na^+^ absorption • NHE1 expression was inhibited • Down-regulated in colon • Up-regulated in colon • Up-regulated in colon	• Shiga toxin 1 induced diarrhea • Rotavirus induced diarrhea in children • UC, CD • UC, CD • DMH-induced CRC	Laiko et al., 2010 Lorrot and Vasseur, 2007; Chen H. et al., 2017 Khan et al., 2003; Sullivan et al., 2009 Khan et al., 1998; Farkas et al., 2011 Goodison et al., 1999; Yonemura et al., 1999; Bourguignon et al., 2004
NHE1	Liver	• Up-regulated • Up-regulated • NHE1 correlates with tumor size, invasiveness and tumor progression	• NAFLD • Hepatic ischemia injury • HCC	Prasad et al., 2013 Levitsky et al., 1998; Wang et al., 2003 Yang et al., 2010; Yang et al., 2011
NHE1	Pancreas	• Locally acidic extracellular conditions favor tumor cell invasion	• PDAC	Rakonczay et al., 2006; Ivanov, 2008; Olszewski and Hamilton, 2009; Olszewski et al., 2010
NHE2	Stomach	• Completion of cell migration during gastric epithelial restitution • Trefoil factor peptides in mouse gastric epithelial restitution requires NHE2 activity • Parietal cell differentiation and longevity	• Gastric epithelial repair • Gastric epithelial repair • NHE2 deficiency is accompanied by a strong, but not total loss of parietal cells the murine stomach	Paehler Vor der Nolte et al., 2017 Xue et al., 2011; Matthis et al., 2019 Schultheis et al., 1998a; Boivin et al., 2000
NHE2	Intestine	• NHE2 activity inhibited • Down-regulated • Mediates only butyrate- dependent Na^+^ absorption	• Cholera toxin induced diarrhea • TNBS-induced colitis in rats • DSS-induced ulcerative colitis	Subramanya et al., 2007 Soleiman et al., 2017 Rajendran et al., 2015
NHE3	Intestine	• The major Na^+^ importer	• ETEC induced diarrhea	Hecht et al., 2004
		• NHE3 activity and protein expression was inhibited	• Cholera toxin induced diarrhea	Subramanya et al., 2007
		• Sodium absorption and acid secretion in human colon	• *Clostridium difficile* induced antibiotic-associated diarrhea	Hayashi et al., 2004; Engevik et al., 2015
		• Down-regulated	• IBD in patients or mice	Sullivan et al., 2009; Yeruva et al., 2010; Farkas et al., 2011; Lenzen et al., 2012, 2018
NHE3	Liver	• Decreased expression of NHE3 in cholangiocytes	• Cholestasis and liver fibrosis	Roussa et al., 2006
NHE3	Gallbladder	• Increased level of NHE3 phosphorylated at serine-552	• Cholesterol gallstone	Chen Y. et al., 2017
NHE3	Pancreatic duct	• In murine pancreatic ducts CFTR controls expression and regulates activity of NHE3	• NHE3 contributes to abnormal pancreatic secretion in cystic fibrosis in mice	Ahn et al., 2001
NHE4	Stomach	• In the basolateral membrane of parietal cells, the differentiation of gastric epithelial cells and the secretion of gastric acid	• NHE4 deficiency in mice causes a decrease in parietal cell number, a loss of mature chief cells, and an increase in the number of mucous and undifferentiated cells	Gawenis et al., 2005
NHE4	Intestine	• NHE4 activity can be reduced	• *Escherichia coli* heat-stable enterotoxin induced diarrhea	Beltrán et al., 2015
NHE6	Intestine	• NHE6 expression was inhibited	• Rotavirus induced diarrhea in children	Lorrot and Vasseur, 2007; Chen H. et al., 2017
NHE8	Stomach	• In the apical membrane of the stomach’s surface mucous cells, bicarbonate secretion and gastric epithelial repair	• NHE8 deficiency causes a decrease in gastric mucosal surface pH and an increased incidence of gastric ulcer	Xu et al., 2013
NHE8	Intestine	• Mucosa protection, mucus secretion	• NHE8 deficiency causes increased inflammation/inflammatory cytokines	Xu et al., 2012; Wang et al., 2015
NHE8	Intestine	• Controls Wnt/β-catenin signaling and Lgr5 expression	• NHE8 deficiency promotes CRC	Xu et al., 2019
NHE9	Intestine	• Up-regulated	• CRC	Ueda et al., 2017
NHA2	Pancreas	• Clathrin-mediated endocytosis and insulin secretion in β-cells	• Pathological glucose tolerance with diminished insulin secretion	Deisl et al., 2013

## NHEs in Esophageal Epithelial Pathology

### The Role of NHEs in Esophageal Injury and Repair

Gastroesophageal reflux disease is characterized by the destruction of the esophageal mucosa caused by uncontrolled reflux of gastric acid, which eventually leads to the formation of esophageal ulcers (Zadeh et al., 2018). Due to the fact that NHE1 expression at mRNA level was detected in both rat and rabbit esophagus, it was assumed that NHE1 may be associated with cytoplasmic pH regulation in esophageal cells and thus contributes to the esophageal defense against the gastric acid reflux (Shallat et al., 1995). To investigate a possible, protective impact of NHE1 on those esophageal cells that are exposed to an extremely acidic environment caused by gastric acid reflux, normal, primary esophageal epithelial cells were cultured and then exposed to an acidic medium of pH 4.0 for up to 12 h. Under these acidic conditions, NHE1 inhibition led to a significant decrease in cell viability suggesting that NHE1 indeed has the ability to protect esophageal cells against the detrimental effects of gastric acid (Park et al., 2015). In this scenario, NHE1 activity is thought to be stimulated by the PKC pathway and Ca^2+^/calmodulin (Fujiwara et al., 2006) which could be mediated by EGF released from the salivary glands (McGurk et al., 1990; Kongara and Soffer, 1999). On the contrary, based on the presence of NHE1 in the basolateral membrane of the esophageal epithelium (Shallat et al., 1995), Siddique and Khan (Siddique and Khan, 2003) argue that the protons extruded by NHE1 lead to a basolateral acidification of the extracellular space which could then cause tissue damage and eventually GERD. They found that NHE1 expression was up-regulated in GERD patients in a histamine-dependent manner via H_2_ receptors. Consequently, they consider NHE1 as a potential therapeutic target and suggest to inhibit NHE1, e.g., by H_2_-receptor blockers, in order to treat GERD (Siddique and Khan, 2003). However, as further explained in the following section, in ESCC, NHE1 has been found to act as a tumor suppressor and to be of prognostic value (Ariyoshi et al., 2017).

### The Role of NHE1 in Barrett’s Esophagus and Its Progression to Esophageal Tumorigenesis

Barrett’s esophagus represents a pre-malignant condition in which original, normal squamous epithelial cells of the distal esophagus have been replaced by columnar-lined intestinal cells. Over time, this so-called intestinal metaplasia tends to become esophageal adenocarcinoma (Bujanda and Hachem, 2018). Although the exact pathological mechanism leading to Barrett’s esophagus is unclear, it is likely to be caused by long-term chronic reflux of gastric or even bile acid and digestive enzymes. Goldman et al. (2010) reported that, compared to its expression in normal esophageal epithelium and normal esophageal cells, NHE1 is highly expressed not only in biopsies from patients with Barrett’s esophagus, but also in the CP-A cell line derived from Barrett’s esophagus. Due to the frequent acid exposure of esophageal cells over a long period, the progressive intestinal metaplasia occurring in Barrett’s esophagus may represent an adaptive process (Ostrowski et al., 2007), which is accompanied by an increase in NHE1 expression in order to defend intracellular pH.

Both bile acids and extracellular acidification, particularly in combination, stimulate the activity of the cytosolic nitric oxide synthase (NOS) and thus nitric oxide (NO) production (Goldman et al., 2010). NO can inhibit NHEs (Coon et al., 2008), which leads to a substantial cytosolic acidification causing DNA damage and subsequent tumor progression (Goldman et al., 2010). On the other hand, Laczko et al. (2016) found that upon chronic exposure to bile acids, NHE1 expression was up-regulated in the Barrett’s esophageal cell lines CP-A and CP-D, at both mRNA and protein level. Besides, it was found that NHE1 activity but not NHE1 expression was increased by acid pulse stimulation in *ex vivo* Barrett’s esophagus tissue, and that the elevated NHE1 activity may be responsible for the hyperproliferation of Barrett’s esophagus epithelial cells, probably mediated by PKC (Fitzgerald et al., 1998). So far, neither the elicitor nor the mechanistic reasons for the differential expression of NHE1 between healthy esophageal epithelium/cells and Barrett’s esophagus epithelial cells are fully understood, and further research focusing on this topic is required.

Esophageal adenocarcinoma (EAC) and ESCC are two main subtypes of esophageal cancer. While sharing a poor 5-year overall survival rate of about 15%, EAC and ESCC originate from different cells and also differ regarding their incidence and epidemiology (Talukdar et al., 2018). EAC is an aggressive malignancy whose occurrence can be significantly abetted by gastric or even bile acid reflux due to GERD (Stephens et al., 2018).

Guan et al. (2014) found that NHE1 was highly expressed in EAC tissue but not in normal esophageal epithelium. Inhibiting the expression of NHE1 with shRNA or its activity with amiloride resulted in an intracellular acidification of esophageal cancer cells which decreased their viability and even induced apoptosis. The suppression of tumor xenograft formation in nude mice could also be observed (Guan et al., 2014). Interestingly, Goldman and colleagues reported that stimulation of JH-EsoAd1 EAC cells or cells of the Barrett’s esophagus cell line CP-A with hydrophobic bile acid will activate NHE. This leads to an increase in the cytosolic Na^+^ concentration which drives K^+^-efflux and decreases the driving force for Na^+^-dependent Ca^2+^-extrusion, resulting in an increased cytosolic Ca^2+^-concentration accompanied by apoptosis (Goldman et al., 2011). There were, in fact, no obvious changes in intracellular pH implying that the ionic imbalance, particularly the increase in the cytosolic Ca^2+^ concentration, was more important than intracellular acidification in inducing apoptosis. Nevertheless, further studies should clarify the role of Na^+^ and H^+^ in the apoptosis of EAC.

Contrary to any expectations, in ESCC cell lines, NHE1 gene knockdown was found to promote proliferation, migration, and invasion, and to inhibit apoptosis via the activation of PI3K-AKT signaling pathway (Ariyoshi et al., 2017). In fact, NHE1 expression was significantly higher in well-differentiated ESCC tumors compared to that in poorly differentiated ESCC tumors and correlated strongly with the 5-year survival rate being much better in the group with high NHE1 expression. Therefore and surprisingly, given the multitude of publications and cancer types in which NHE1 expression/activity positively correlates with malignancy, NHE1 could be of prognostic value in ESCC.

## NHEs and Gastric Epithelial Pathology

### NHEs and Gastric Epithelial Repair

The integrity of the gastric epithelium is essential for the protection against gastric luminal contents such as gastric acid (pH 1-2), pepsin and bacteria. Gastric mucosal injury, when including the epithelium, will initiate the migration of cells located close to the wound area. Different NHE isoforms have been shown to be involved in both maintaining the integrity of the gastric epithelium and gastric epithelial cell migration. In rabbit gastric epithelium NHE1, 2, and 4 are expressed. NHE1 and NHE2 are the predominant isoforms in mucous cells where they regulate intracellular pH, especially during high gastric acidity, and NHE4 expression is higher in parietal and chief cells where it contributes to volume regulation during gastric acid secretion (Rossmann et al., 2001). Paehler Vor der Nolte et al. (2017) reported that the migration of rat gastric surface cells (RGM1) is promoted by NHE1 activation, however, inhibited by activation of NHE2. Therefore, the authors supposed that NHE1 may be responsible for the initiation while NHE2 contributes to the completion of cell migration during gastric epithelial restitution. However, based on a series of studies on NHE2 knockout mice, the team of Marshall Montrose reported that NHE2, but not NHE1, is essential for mouse gastric epithelial repair, and that the enhancing effect of trefoil factor peptides in mouse gastric epithelial restitution requires NHE2 activity (Xue et al., 2011; Matthis et al., 2019). Furthermore, NHE2 may be involved in cell differentiation and longevity because its deficiency is accompanied by a strong, but not total loss of parietal cells in the murine stomach (Schultheis et al., 1998a; Boivin et al., 2000). Thus, NHE2 can be considered essential for the formation and maintenance of the normal gastric epithelium (Aihara et al., 2016). Interestingly, NHE1-3 expression was detected also in human gastric myofibroblasts. In these cells, NHE1 is necessary for insulin-like growth factor II-induced proliferation and involved in carbachol- and insulin-like growth factor II-stimulated migration (Czepán et al., 2012). NHE4 is located in the basolateral membrane of parietal cells. Its gene deficiency in mice causes (i) a decrease in the number of parietal cells, (ii) a loss of mature chief cells, and (iii) an increase in the number of mucous and undifferentiated cells (Gawenis et al., 2005). So, similar to NHE2, also NHE4 is involved in the differentiation of gastric epithelial cells and the secretion of gastric acid. NHE8 is found in the apical membrane of the stomach’s surface mucous cells. In mice, NHE8 gene deficiency has no effect on the secretion of basal gastric acid (Xu et al., 2013). However, NHE8 deficiency causes a decrease in gastric mucosal surface pH and an increased incidence of gastric ulcer. In addition, the expression of the Cl^–^/HCO_3_^–^ exchanger DRA (downregulated-in-adenoma) is suppressed in NHE8 knockout mice (Xu et al., 2013). These above-mentioned observations imply that NHE8 is associated with bicarbonate secretion and gastric epithelial repair.

### NHEs and Gastric Cancer

Due to the important role of NHE family members, especially NHE1, in tumorigenesis and tumor progression (Stock and Schwab, 2015; Stock and Pedersen, 2017), the role of NHEs in the pathophysiology of gastric cancer has been examined. NHE1 expression is significantly higher in both human gastric cancer tissue and cells when compared to that in normal gastric tissue and cells, and the inhibition of NHE1 suppresses proliferation, migration, and invasion of gastric cancer cells (Xie et al., 2017). In order to clarify the detailed mechanism by which NHE1 affects the pathophysiology of gastric cancer, various gastric cancer cell models were used. Hosogi et al. (2012) found that, when human gastric cancer cells (MKN28) are stimulated with the NHE-inhibitor EIPA, the intracellular pH does not change because of a compensatory increase in HCO_3_^–^-uptake mediated by the Na^+^-driven Cl^–^/HCO_3_^–^ exchanger NDCBE (Hosogi et al., 2012). The concomitant decrease in the cytosolic Cl^–^ concentration is thought to activate MAPK and enhance p21 expression, which then leads to G_0_/G_1_ arrest and reduced proliferation in MKN gastric cancer cells (Hosogi et al., 2012). In human gastric carcinoma cells (SGC-7901), knocking down NHE1 causes a decrease in cell proliferation, G1/G0 phase arrest, increased apoptosis, and, when SCG-7901 cells are injected subcutaneously into nude mice, a reduced tumorigenic capacity (Liu et al., 2008). Moreover, in human gastric cancer cells of the MKN45 and the MKN74 line, inhibition of NHE1 activity by 2-amino-3*H*-phenoxazin-3-one (questiomycin A), a derivative of the oxidative phenoxazine, leads to a decrease in pH_i_ and promotes apoptosis (Nagata et al., 2011). Altogether, inhibition of NHE1 causes changes in the ionic composition of the cytosol that are likely to contribute to the pathogenesis of gastric cancer in terms of cell proliferation, cell apoptosis, and cell cycle. In fact, NHE1 may act as a link between ion channels and tumorigenic signaling because it can be regulated through the NF-κB signaling pathway depending on both the expression and also the activity of the voltage-gated sodium channel Na_v_1.7. Na_v_1.7 is found to be abundantly expressed in both gastric cancer tissue and the gastric cell lines BGC-823 and MKN-28 (Xia et al., 2016).

It has been known that overexpression of VEGF is critical for tumor formation and vascularization. In human gastric cancer cells (SGC7901), a decrease in pH_i_ induced by NHE1 inhibition is accompanied by an increase in VEGF expression at both mRNA and protein level (He et al., 2010). This illustrates the role of NHE1 in the pathophysiology of gastric cancer once more. There may be even more mechanisms to be elucidated by which NHE1 and the triggering or progress of gastric cancerogenesis could be interwoven.

## NHEs and Intestinal Disease

### The Function of NHE in Intestinal Inflammation and Diarrheal Disease

Intestinal Na^+^ absorption is mastered mainly by NHEs so that NHE inhibition will result in the reduction of Na^+^ absorption accompanied by osmotic or even secretory diarrhea (Gurney et al., 2017). Enterotoxigenic *Escherichia coli* (ETEC) are one of the most common pathogens known to cause intestinal diarrhea (Gupta et al., 2008; Maigaard Hermansen et al., 2018). When intestinal epithelial cells of the Caco-2 line are stimulated with EPEC, the total activity of all NHEs in their entirety is increased. This impact on NHE activity requires the type III secretion system (TTSS), a complex multiprotein structure produced by some gram-negative bacteria that enables them to inject their prokaryotic molecules into the eukaryotic host cell. Interestingly, EPEC infection leads to a significant increase in the basolateral NHE1 activity, a ∼300% increase in apical NHE2 and a 50% decrease in apical NHE3 activity (Hecht et al., 2004). The massive decrease in the activity of NHE3 as the major Na^+^ importer, probably combined with an EPEC-mediated decrease in apical Cl^–^/HCO_3_^–^ exchange, is thought to contribute, at least in part, to the pathophysiological basis of EPEC-induced diarrhea (Hecht et al., 2004).

Shiga toxin 1 from enterohemorrhagic *Escherichia coli* causes a depletion of galectin-3 which leads to mistargeting of apical proteins in T84 cells. In addition to villin and dipeptidyl peptidase IV, also NHE2, one of the major colonic Na^+^ absorptive proteins, is mistargeted into basolateral compartments. Consequently, the resulting failure of NHE2-mediated Na^+^ absorption will contribute to diarrhea (Laiko et al., 2010).

Also in T84 cells, the activity of NHE4 can be reduced by *Escherichia coli* heat-stable enterotoxin via up-regulation of cAMP and PKA activity. Beltrán et al. (2015) speculate that the subsequent intracellular acidification may induce an increase in the conductance of the apically located CFTR accompanied by an increased Cl^–^ efflux into the intestinal lumen and, thus, promotes the pathogenesis of diarrhea.

The opening probability of the Cl^–^ conducting CFTR is strongly increased by cholera toxin. Basically, the cholera toxin causes the adenylyl cyclase to be permanently highly active, and the continuously high cAMP level stimulates Cl^–^ efflux through CFTR, thus breeding and feeding secretory diarrhea (Islam et al., 2018). In addition to this tremendous increase in Cl^–^ secretion the cholera toxin causes a considerable decrease in the apical Na^+^ resorption. In rat ileum treated with cholera toxin *in vivo*, the total NHE activity was reduced by 70%. NHE2 activity, but not its expression, was inhibited while both activity and protein expression of NHE3 were inhibited by the cholera toxin (Subramanya et al., 2007).

*Clostridium difficile* is primarily responsible for antibiotic-associated diarrhea coming along with reductions in NHE3 expression, Na^+^ absorption and acid secretion in human colon (Engevik et al., 2015). In kidney epithelial and placental cell lines, the group of John Orlowski and Sergio Grinstein observed a retranslocation of the apically located NHE3 to subapical endomembrane compartments in response to the *Clostridium difficile* toxin B (Hayashi et al., 2004). They propose that clostridial toxin B induces a change in the ezrin-mediated interaction between NHE3 and the microvillar cytoskeleton via inactivation of Rho-family GTPases (Hayashi et al., 2004).

Rotavirus is the leading etiology of acute watery diarrhea in children and one of common causes of death in children under the age of 5 years (Operario et al., 2017; Tian et al., 2018). Although the rotavirus inhibits the expression of the basolaterally located NHE1 and the organellar NHE6 in Caco-2 cells (Chen H. et al., 2017), it is mainly the rotavirus nonstructural protein NSP4 that causes diarrhea by inhibiting SGLT (Na^+^, glucose-cotransporter)-mediated Na^+^- and glucose-absorption and by increasing both the paracellular permeability and the phospholipase/IP3-controlled cytosolic Ca^2+^ concentration ([Ca^2+^]_i_). An increase in [Ca^2+^]_i_ then stimulates luminal Cl^–^ secretion (Lorrot and Vasseur, 2007).

### Intestinal NHEs in IBD

Inflammatory bowel disease is a chronic inflammatory disorder which is classified into UC and CD (Arnott et al., 2018). One of the common symptoms in IBD patients is diarrhea, possibly caused by NHEs’ dysfunction (Magalhaes et al., 2016). Compared to healthy probands, NHE1 expression in colonic biopsies from both UC and CD patients is reduced at mRNA and protein level (Khan et al., 2003). Also, Sullivan et al. reported that NHE1 is down-regulated in the colon from patients who suffer from UC or CD, and in the colon from DSS- and TNBS-induced IBD mouse models (Sullivan et al., 2009). In contrast, Khan et al. (1998) found that NHE1 expression at mRNA and protein level is significantly increased in acetic acid- and TNBS-induced colitis in Sprague-Dawley rats. Furthermore, Farkas et al. (2011) reported that the activity of NHE1 including its mRNA expression are significantly upregulated in the colonic crypts isolated from UC patients compared to those from healthy human. These contradictions are probably due to pathophysiological differences between IBD animal models and among patients (Khan et al., 2003).

NHE2 is located mainly in the apical membrane of colonic epithelial cells. Its deficiency does not lead to intestinal diarrhea in mice (Schultheis et al., 1998b; Ledoussal et al., 2001). In colon biopsies from IBD patients, NHE2 expression and activity are not significantly changed (Sullivan et al., 2009; Farkas et al., 2011). Given the multitude of proinflammatory cytokines secreted under the pathological condition of IBD, both TNF-α (tumor necrosis factor α) and IFNγ (interferon γ) could suppress NHE2 expression and activity in the intestinal epithelial cells through NF-κB activation (Rocha et al., 2001; Amin et al., 2011). Furthermore, Soleiman et al. (2017) reported that in colon from Sprague-Dawley rats with TNBS-induced colitis NHE2 expression is down-regulated at both mRNA and protein level, which implies that NHE2 is indeed involved in the pathogenesis and pathophysiology of IBD. Surprisingly, Rajendran et al. (2015) found that neither NHE2 nor NHE3 are altered in the inflamed colon obtained from Sprague-Dawley rats with DSS-induced UC compared to the normal colon from untreated specimens. In addition, NHE3 conducts both HCO_3_^–^-dependent and butyrate-dependent Na^+^ absorption under normal physiological conditions, while NHE2 is activated by DSS-induced UC and mediates only butyrate-dependent Na^+^ absorption in the inflamed colon (Rajendran et al., 2015). Since NHE2 is active mainly under pathophysiological conditions, targeting the mechanism of butyrate activation of NHE2 could be a novel therapeutic strategy to treat diarrhea in IBD.

NHE3 is primarily located in the surface area of colonic crypts and its functional deficiency contributes significantly to the diarrheal syndrome in IBD (Larmonier et al., 2011). Sullivan et al. (2009) reported that NHE3 protein expression is down-regulated in mucosal biopsies taken from the sigmoid colon of IBD patients as well as in the colon from mice with DSS- and TNBS-induced colitis. Furthermore, it was revealed that in the intestinal epithelial cells of the C2BBe1 line both IFNγ and TNF-α can inhibit NHE3 expression via PKA-mediated phosphorylation of the transcription factors Sp1 and Sp3 (Rocha et al., 2001; Amin et al., 2006). Based on these observations, the decrease in NHE3 protein expression could be considered a possible reason for IBD-associated diarrhea. On the other hand, however, Farkas et al. (2011) found in colonic crypts isolated from UC patients and healthy controls that, while NHE3 activity was indeed inhibited in the colon from UC patients compared to that from healthy controls, NHE3 mRNA expression remained unchanged. Similarly, Yeruva et al. (2010) reported that although neither NHE3 expression nor its localization in the brush border membrane was changed in sigmoid colon from UC patients, NHE3 activity including Na^+^ absorption were reduced significantly. Also in colons of IL-10 (interleukin 10) deficient mice and in mice with DSS-induced colitis NHE3 activity is down-regulated whereas its expression and membrane localization are not affected. In search of the mechanism by which NHE3 activity is reduced Lenzen et al. (2012) found that both mRNA and protein expression of PDZK1, a scaffold protein forming a submembranous signal complex together with NHE3, are down-regulated which may be the possible reason for the dysfunction of NHE3 in IBD. Furthermore, DSS-induced colitis in mice is accompanied by high expression of pro-inflammatory cytokines such as interleukin IL-1β and TNF-α, the loss of NHE3 and PDZK1, and a decreased fluid absorption in colon. The pathological conditions mentioned above could be counteracted by a concomitant anti-TNF-α treatment resulting in both the restoration of the NHE3 and PDZK1 location in the apical region of enterocytes and the normalization of NHE3-mediated fluid absorption (Lenzen et al., 2018). This finding further confirms the role of PDZK1 in NHE3 (dys)function and subsequent diarrhea in IBD.

### The Impact of NHEs on Gut Microbiota and Intestinal Mucosal Integrity

The homeostasis of gut microbiota is essential for the integrity of the intestinal mucosa and the maintenance of the intestinal microenvironment. An alteration of the gut microbiota can lead to various intestinal diseases, such as IBD (Nishida et al., 2018), *Clostridium difficile* infection (De Wolfe et al., 2018), irritable bowel disease (Stern and Brenner, 2018), and others. NHE-mediated alterations in the intestinal microenvironment, such as changes in luminal ion concentration and pH, could damage the gut microbiota. For instance, gram-positive bacteria such as *Clostridium* and *Lactobacillus* are increased in cecum and colon of NHE2 knockout mice although the total luminal and mucosa-associated bacteria remain unchanged (Engevik et al., 2013b). Also NHE3 knockout has an impact on the murine gut microbial environment, which includes a decrease in *Lachnospiraceae* and *Ruminococcaceae* (Firmicutes) and an increase in *Bacteroidaceae*. These changes in the microbiome then lead to the initiation and intensification of colitis. Hence, disruption of NHE3 function in gut may modulate the pathogenesis and progression of colitis via alteration of the microbial ecology (Harrison et al., 2018). In addition, NHE3 deficiency reinforces the T-cell mediated immune response to a disturbed gut microbiome (Laubitz et al., 2016). Consequently, NHE3 plays an important role in keeping the gut’s microbial ecology balanced, and NHE3 malfunction caused by inflammatory cytokines results in the onset and fuels the progress of the IBD. Furthermore, in NHE3-deficient mice, the luminal ion composition and pH are altered locally leading to region-specific changes in the composition of the bacterial population (Engevik et al., 2013a). Compared to NHE3 wild-type mice, the diversity of the luminal and mucosal microbiota in NHE3 knockout mice is significantly decreased (Larmonier et al., 2013), and the bacterial composition in their intestine affects the severity of colitis (Larmonier et al., 2013). In summary, NHE3 is essential for the generation and maintenance of an intact microbiota, because NHE3 deficiency is accompanied by the pathogenesis of IBD, probably caused by a disproportion in the bacterial composition.

NHE8 contributes significantly to intestinal mucosa protection. In NHE8 knockout mice the expression of mucin 2, an important component of the mucus layer, is clearly decreased (Xu et al., 2012). The mucus layer coats the intestinal epithelium and represents a defense mechanism against both commensal and pathogenic bacteria by separating the apical membranes of intestinal epithelial cells from the luminal contents. In DSS-treated NHE8 knockout mice, the low mucin production in goblet cells and a reduced production of enteric defensins, due to decreases in both the number of mature goblet cells and the population of functional Paneth cells, indicate that NHE8 contributes to the intestinal mucosal integrity through goblet and Paneth cells. According to this, the DSS-treated NHE8 knockout mice are characterized by increased inflammation and elevated expressions of IL-1β, IL-6, IL-4, and TNF-α (Wang et al., 2015).

In their entirety, the above-mentioned findings suggest that NHEs have a strong impact on the gut microbiota and the integrity of the intestinal mucosa, and that NHE dysfunction can be associated with the pathogenesis of various intestinal diseases.

## NHE and Colon Cancer

Intracellular acidification is a trigger in the early phase of apoptosis and results in activation of endonucleases inducing DNA fragmentation, whereas alkalinization of the cytosol is an early event in malignant transformation and plays an essential role in tumor progression (Izumi et al., 2003). In solid tumors, cellular pH regulators, particularly NHE1, are up-regulated, and the resulting intracellular alkalinization and extracellular acidification play crucial roles in cell proliferation, invasion and metastasis (Reshkin et al., 2000). Accordingly, NHE1-deficient cells show no or a strongly reduced tumor growth *in vivo* (Rotin et al., 1989). Furthermore, cells lacking NHE activity exhibit a markedly impaired proliferation, indicating that NHE activity is important for tumor growth and cell proliferation (Izumi et al., 2003). A recent study by Stine Pedersen’s lab demonstrates how both the intracellular steady state pH and NHE1 expression vary over the different phases of the cell cycle (Flinck et al., 2018). The authors propose that cell cycle phase-specific regulation of the intracellular pH_i_ is controlled by NHE1 and the Na^+^, HCO_3_^–^ -cotransporter NBCn1. On the whole, NHE1, the activity of which is triggered by cellular acidosis and cell shrinkage, has an anti-apoptotic effect (Lang et al., 2000b). This is consistent with the observation that activating the Fas receptor - which can induce apoptosis - inhibits NHE1 activity in T-lymphocytes via activation of the Src-like kinase Lck^56^ (Lang et al., 2000a). Conversely, mitogen- or growth factor-induced phosphorylation of specific regions of NHE1’s regulatory C-terminus is essential for NHE1 activity and can counter both cell shrinkage and intracellular acidification. NHE1 interacts with proteins of the ezrin, radixin, moesin (ERM) family (Denker et al., 2000) and also with CD44 (Bourguignon et al., 2004), an adhesion molecule which (i) is also known to interact with ERM proteins, (ii) is ubiquitously expressed in cancer cells and (iii) regulates cell adhesion and motility (Goodison et al., 1999; Yonemura et al., 1999). Even the formation of tumor cell pseudopodia is regulated by NHE1, on the one hand via its interaction with F-actin-associated proteins, on the other hand by local pH regulation (Magalhaes et al., 2011; Ludwig et al., 2013).

To date, there are only a few studies reporting on the involvement of NHEs in colon cancer. Vaish and Sanyal found in rats that the protein expression level of NHE1 is significantly higher in DMH-induced CRC tissue than in colorectal tissue of the control group. Co-administration of DMH and non-steroidal anti-inflammatory drugs (NSAIDs) such as the CRC-preventing sulindac and celecoxib leads to significant decreases in NHE1 expression implying that NHE1 contributes to colonic tumor progression (Vaish and Sanyal, 2012). Both the activity and the expression of NHE1 are increased also in doxorubicin-resistant (HT29-dx) human colon carcinoma cells suggesting a role for NHE1 in the development of multidrug resistance in tumor cells (Miraglia et al., 2005). NHE1-mediated alkalinization of the cytosol seems to facilitate Wnt/β-catenin signaling in human colorectal tumor cells (Serafino et al., 2012). Constitutive Wnt/β-catenin signaling is accompanied by redistribution of β-catenin into the nucleus and a decreasing expression of E-cadherin. In epithelial cells, low or even no *E*-cadherin expression is an indicator for malignant transformation. Serafino et al. (2012) discuss Akt-activity as the possible mechanistic link between NHE1-modulated cytosolic pH and Wnt/β-catenin signaling.

An increased Wnt/β-catenin signaling is found also in the absence of NHE8 (Xu et al., 2019). The absence of NHE8 most likely makes a considerable contribution to the development of intestinal tumors, because it is expressed in normal human colon tissue, but cannot be detected in CRC. Furthermore, in the azoxymethane/dextran sodium sulfate cancer model, only 9% of NHE wild-type mice displayed tumorigenesis as opposed to 89% in NHE8 knockout mice. Finally, NHE8 deficiency is accompanied by an elevated expression of the stem cell marker Lgr5 in the colon (Xu et al., 2019).

Recently, the physiological function of NHE9 has been widely investigated in various human cell lines and tissues. Ueda et al. (2017) revealed by immunohistochemical analysis that NHE9 is up-regulated in CRC compared to normal tissue. NHE9 is closely related to EGFR (EGF receptor) signaling pathways. Its overexpression promotes the progression and the metastatic potential of CRC and, thus, may be a prognostic marker and a potential therapeutic target (Ueda et al., 2017).

## Role of NHEs in Hepatic Ischemia and Liver Cancer

Increased Na^+^ influx via ion transporters including Na^+^/H^+^ exchangers is required for hepatocyte proliferation (Koch and Leffert, 1979). As reported by Forgac and Arias, an amiloride-sensitive Na^+^/H^+^ exchanger is situated in the sinusoidal domain of the plasma membrane of rat hepatocytes and probably involved in the regulation of pH_i_ (Arias and Forgac, 1984). In primary cultures of hepatocytes treated with EGF, the increase in pH_i_ is indeed mediated by the Na^+^/H^+^ exchanger whose activation is tyrosine kinase-dependent and regulated by modulations of the cytosolic Ca^2+^ concentration including Ca^2+^-calmodulin-dependent pathways (Tanaka et al., 1994). NHE activity is significantly higher in basolateral plasma membrane vesicles isolated from neonatal rat liver than in those from adults. This is due to an increased number or an increased individual activity of antiporters in the neonatal plasma membrane (Goodrich and Suchy, 1990).

Hepatic stellate cells, also known as perisinusoidal cells, represent the major cell type involved in liver fibrosis. Quiescent stellate cells constitute 5-8% of the total number of liver cells. HSCs can become activated when the liver is damaged. An activated HSC is characterized by proliferation, contractility, and chemotaxis. In this state, HSCs are the main source of extracellular matrix production in the context of liver injuries and also responsible for secreting collagen into the scar tissue which, eventually, can result in cirrhosis. Na^+^/H^+^ exchange is the main regulator responsible for pH_i_ homeostasis in rat HSCs. Activation of HSCs is associated with an increased NHE activity accompanied by an elevated steady state pH_i_, and it also contributes to proliferation in response to stimulation by the platelet derived growth factor (PDGF) (Di Sario et al., 1997). Activation of NHE in HSCs occurs also in oxidative stress-associated liver fibrosis, implying that the Na^+^/H^+^ exchange might act as a common mediator of diverse effects induced by oxidative stress. Moreover, the NHE inhibitor amiloride can suppress HSC proliferation and collagen secretion which suggests NHE to be a potential therapeutic target in the treatment of liver fibrosis (Svegliati-Baroni et al., 1999). Similarly, *in vivo* findings showed that liver fibrosis induced by dimethylnitrosamine or bile duct ligation can be significantly ameliorated by simultaneous administration of amiloride (Haussinger, 2001).

Development of fibrosis is crucial in the pathogenesis of NAFLD (Rombouts and Marra, 2010; Takaki et al., 2013), and NHE activity might also be involved in NAFLD induced by HFD. Compared to WT mice with a normal chow diet, livers of mice with HFD displayed a nearly twofold increase in NHE1 expression, suggesting a potential role of NHE activity in different stages of NAFLD (Prasad et al., 2013). Long-term ablation of NHE1 activity in mice attenuates HFD-induced lipid accumulation in liver and preserves insulin sensitivity (Prasad et al., 2013). Strong inhibition of NHE1, present not only in the plasma membrane but also in the mitochondrial inner membrane, may as well have protective effects in chronic liver diseases leading to steatosis and fibrosis because it is accompanied by a decrease in ROS production, i.e., an indirect increase in antioxidant potential, and an improved mitochondrial efficiency in energy conversion (Javadov et al., 2011; Alvarez and Villa-Abrille, 2013). Furthermore, like in myocardial ischemia, NHE1-inhibition by EIPA reduces oxidative liver damage caused by reperfusion after ischemia in a rat model (Wang et al., 2003). Ischemic conditions impede the end-oxidation in the mitochondria which leads to an accumulation of H^+^ ions. This decline in cytosolic pH stimulates NHE1 activity resulting in an increased cytosolic Na^+^ concentration accompanied by a decrease in the driving force for the Na^+^/Ca^2+^ exchanger. The consequential elevation of the cytosolic Ca^2+^ concentration modulates or inhibits enzyme activities and even induces apoptosis (Levitsky et al., 1998).

In rat hepatocytes, agents promoting liver tumors, such as α-hexachlorocyclohexane or phenobarbital, cause an intracellular alkalinization mediated by Na^+^/H^+^ antiport. A higher cytosolic pH then stimulates DNA synthesis which is an important event in malignant transformation and tumor progression (Lee et al., 2003). In HCC tissues, increased NHE1 expression levels correlate with tumor size, the ability of invasiveness and tumor progression (Yang et al., 2011). *In vitro*, inhibition of NHE1 expression via siRNA-mediated knockdown reduces HCC growth and induces apoptosis. Moreover, treatment with EIPA suppresses tumor growth in nude mouse xenografts of HCC cells (Yang et al., 2010), suggesting that NHE1 might be a potential therapeutic target in HCC.

Cholangiocytes represent the cuboidal epithelium in the small interlobular bile ducts and contribute to bile secretion via net release of HCO_3_^–^ and H_2_O. NHE3 is expressed in the luminal membranes of murine and rat cholangiocytes where it plays a role in fluid secretion and absorption from the luminal space (Mennone et al., 2001). Cultured cholangiocytes isolated from patients with primary biliary cirrhosis exhibit impaired functions of the Cl^–^/HCO_3_^–^ anion exchanger 2 (AE2) and amiloride-sensitive NHE(s). Moreover, the insensitivity of AE2 and NHE to secretory stimuli results in the inability of the biliary epithelium to alkalinize and hydrate the bile, which consequently aggravates the cholestatic condition and at the same time may be a molecular mechanism underlying, at least contributing to, the impaired HCO_3_^–^ secretion during cholestatic disease (Melero et al., 2002). Bile duct ligation as an experimental model to induce cholestasis and liver fibrosis in rats results in decreased expression of NHE3 in cholangiocytes, which, due to lower Na^+^-(re-)absorption, may contribute to the enhanced salt and fluid secretion of rat livers in response to stimulation with secretin (Roussa et al., 2006). Altogether, the above-mentioned observations strongly support the view that reduced NHE3 activity is responsible for a diminished absorptive capacity in cholangiocytes.

## Physiology and Function of NHEs in Gallbladder

Since in the gallbladder, absorption of water and electrolytes is (i) associated with luminal proton secretion and (ii) inhibitable by amiloride (Weinman and Reuss, 1982; Rege and Moore, 1987), NHEs are believed to play an important role in this process. Early studies demonstrate that in the human gallbladder NHE3 is expressed only in the absorptive epithelial cells and is therefore likely to be the major isoform involved in water and electrolyte absorption from bile (Silviani et al., 1997). In prairie dogs, however, both of the apical isoforms NHE2 and NHE3, but not NHE1, contribute to Na^+^ uptake and transepithelial Na^+^ absorption (Abedin et al., 2001). A rather recent study shows an increased level of NHE3 phosphorylated at serine-552 in response to diet-induced cholesterol gallstone formation in mice. This increase in phosphorylation is thought to lead to a higher turnover of NHE3 resulting in a downturn of the gallbladder’s concentrating function. The other way around, decreased bile absorption by the gallbladder may be the result of decreased exchanger activity due to phosphorylation-induced turnover in murine gallbladder epithelium cells (Chen Y. et al., 2017). Further studies are required to address the underlying mechanisms.

## NHEs in Pancreas

The pancreas is a mixed glandular organ both in the digestive and the endocrine system of vertebrates. It functions as an important endocrine organ by producing several hormones including insulin, glucagon, somatostatin, and pancreatic polypeptide, all of which circulate in the blood. As a digestive organ, the pancreas secretes (i) pancreatic juice containing HCO_3_^–^ to neutralize the acidic chyme arriving from the stomach and (ii) digestive enzymes that facilitate digestion and absorption of nutrients in the small intestine.

In murine islets of the endocrine pancreas eight NHE isoforms are expressed at mRNA level. Among these, two, namely NHE1 and NHE5, are located predominantly in the plasmalemma and six are found intracellularly, including NHA1, NHA2, NHE6, 7, 8, and 9. NHA1 and NHA2 belong to the SLC9B subgroup (SLC9B1 and SLC9B2, respectively) and are also known as NHEDC1 and NHEDC2. To date, just the ubiquitous isoform NHE1 and the recently cloned isoform NHA2 have been fully investigated in β-cells (Moulin et al., 2007; Deisl et al., 2013).

Secretion of insulin by pancreatic β-cells is highly relevant to circulating glucose levels. Glucose uptake into β-cells is mediated by the SLC2 (GLUT) transporter family and leads to the initiation of the insulin secretion cascade (Mueckler and Thorens, 2013). High glucose increases the cytoplasmic pH of primary β-cells and β-cell lines (Lindstrom and Sehlin, 1984; Doliba et al., 2007). In the 1980s, glucose-induced intracellular alkalinization was believed to be the key event in insulin secretion (Lindstrom and Sehlin, 1984). Later, Juntti-Berggren et al. (1991) reasoned that the glucose-induced alkalinization was a result of plasmalemmal NHE activity because it depended on extracellular Na^+^ and was sensitive to the NHE inhibitor EIPA. However, contrary studies showed that insulin secretion was stimulated by NHE inhibition but hindered by intracellular alkalinization (Pace et al., 1983; Best et al., 1988). In order to address this inconsistency, Stiernet et al. (2007) investigated NHE1 mutant mice (NHE1^swe/swe^, swe, slow wave epilepsy). They concluded that NHE1 is the only NHE isoform present in the cell membrane of murine β-cells and activated at low intracellular pH in the absence of HCO_3_^–^/CO_2_. Under physiological conditions, however, the intracellular alkalinization caused by high glucose is entirely dependent on HCO_3_^–^ production (Stiernet et al., 2007). Moreover, NHE1 has no impact on stimulus-secretion coupling, and the NHE inhibitors EIPA and DMA, but not the selective NHE1 inhibitor cariporide, stimulate insulin secretion without pH_i_ changes in both NHE1 wild-type and mutant islets (Stiernet et al., 2007). There is a Na^+^ background current in pancreatic β-cells the nature of which has not been further characterized (Fridlyand et al., 2013). It might well be that EIPA and DMA affect this Na^+^ background current and thus contribute to a depolarization of the membrane potential which is essential for the Ca^2+^-dependent release of insulin.

The role of NHA2 in insulin secretion has been less controversial. The NHA2 protein resides in transferrin-positive endosomes and synaptic-like microvesicles (SLMVs), but not in insulin-containing, large and dense core vesicles (LDCVs) (Deisl et al., 2013). In *in vivo* studies on two different NHA2 knock-out strains, mice were subjected to intraperitoneal glucose and insulin tolerance tests. The experiments revealed a pathological glucose tolerance with diminished insulin secretion, but at the same time a normal peripheral insulin sensitivity. *In vitro* studies on islets isolated from NHA2 knock-out or heterozygous mice demonstrate a deficient insulin secretion after stimulation with glucose or the antihyperglycemic agent tolbutamide (Deisl et al., 2013). The data from Deisl et al. (2013) indicate a pivotal role of NHA2 in both clathrin-mediated endocytosis and insulin secretion in β-cells, suggesting that defective endo-exocytosis coupling may be the mechanism underlying the secretory deficit observed in NHA2-deficient mice. The precise function of NHA2 still remains unclear and future work should address the potential involvement of NHA2 in the pathogenesis of β-cell disorders in humans.

The exocrine part of the pancreas represents up to 90% of the pancreatic mass and is composed of acinar, centroacinar, and ductal cells. The secretory cells of the acini surround small intercalated ducts and contain plenty of small, visible granules of zymogens (Pandiri, 2014). In response to extracellular stimuli, pancreatic acinar cells synthesize, package, and secrete zymogens. They also secrete an isotonic, NaCl-rich, neutral fluid. Ductal cells exchange Cl^–^ for HCO_3_^–^ in order to produce an alkaline fluid, thus providing an optimal pH for digestive enzymes and neutralizing the gastric acid entering the duodenum (Steward et al., 2005; Hegyi et al., 2011). Several NHE isoforms have been detected in the pancreas, although their cellular or organellar distribution and functions have not been fully investigated. NHE1 is ubiquitously expressed and localized to the basolateral membrane of pancreatic ductal epithelial cells (Anderie et al., 1998). It has been observed that Nhe1^–/–^ murine acinar cells fail to recover from an acid load, suggesting that NHE1 is the major isoform responsible for Na^+^/H^+^ exchange (Brown et al., 2003). Additionally, even in the presence of extracellular HCO_3_^–^, acini from Nhe1^–/–^ animals do not recover from an intracellular acid challenge, implying that basolateral Na^+^/HCO_3_^–^ exchange was unable to compensate for the loss of NHE1 to buffer the pH_i_ changes (Brown et al., 2003). In contrast, NHE2 and NHE3 seem to play a minor role in the pancreatic acinar cells since acini isolated from either Nhe2^–/–^ or Nhe3^–/–^ mice showed no difference in the pH_i_ recovery from an acid load when compared to WT mice (Anderie et al., 1998). Nonetheless, NHE3 expression and activity in the luminal membranes of mouse pancreatic ducts are measurably affected by CFTR (Ahn et al., 2001). On the other hand, NHE3 expression could not be confirmed in the human pancreatic ductal carcinoma cell line CFPAC1 (Rakonczay et al., 2008). NHE4 is found to be located in the basolateral membranes of pancreatic acinar and duct cells. NHE1 and NHE4 are also present in membranes of zymogen granula of rat exocrine pancreas (Anderie et al., 1998).

To date, there have been limited reports in respect of the function of NHE in pancreatic diseases. A recent study revealed that the NHE1 inhibitor EIPA inhibits micropinocytosis in PDAC, and micropinocytosis is required for albumin-dependent cancer cell proliferation (Commisso et al., 2013). This observation is consistent with a series of previous findings of the importance of NHE1 activity for endocytotic processes (Ivanov, 2008). Moreover, other studies also showed expression and function of NHE1 in several PDAC cell lines (Rakonczay et al., 2006). It has been found that NHE1 activation by growth factors like neurotensin and its analogs was accompanied by phosphorylation of serine residues of the exchanger, leading to locally acidic extracellular conditions favoring tumor cell invasion at a very early stage of tumor development (Olszewski and Hamilton, 2009; Olszewski et al., 2010). At the same time, NHE1 favors formation of invadopodial structures (Cardone et al., 2015) required for invasion which is consistent with the general idea that an acidic extracellular pH promotes tumor invasion by stimulation of protease secretion and induction of proinvasive mediators, and also by suppression of apoptosis through intracellular alkalinization (Lagadic-Gossmann et al., 2004; Cardone et al., 2005).

## Targeted Therapy

### Modulation of NHE1 Expression/Activity as a Potential Therapeutic Target in Digestive System Malignancy

In recent years an increasing number of publications have emphasized the growing importance of hydrogen ion dynamics in modern cancer research, from etiopathogenesis to treatment. Among all NHE family members, NHE1 is the best studied and has been demonstrated to be directly associated with cellular transformation, proliferation, invasion and metastasis (Loo et al., 2012). As described earlier in this review, NHE1 plays important roles in the tumorigenesis of various digestive organs such as HCC, ESCC, gastric cancer, colon cancer and PDAC. An elevated NHE1 activity, resulting in both an increase in intracellular pH and a decrease in the extracellular pH of tumors, is considered to be the major factor in promoting extracellular/interstitial acidity already at the earliest pre-cancer stage of oncogene-driven neoplastic transformation (Harguindey et al., 2013). Inhibition of NHE1 expression or activity leads to tumor cell growth arrest, acidification of the intracellular space, apoptosis and inhibition of glycolysis (Rich et al., 2000; Che et al., 2011). These observations have boosted the interest in NHE1 as a promising and highly selective drug target in anticancer therapy (Stock et al., 2012). For instance, amiloride, the first NHE inhibitor, was shown to lower VEGF production and to reduce the activity of MMPs, urokinase-type plasminogen activator (μPA), and other proteases, all of which facilitate the initiation and progression of the metastatic process (Kim et al., 2006; He et al., 2007; Provost et al., 2012; Provost and Wallert, 2013). Another study showed that suppression of (i) NHE1 expression by using siRNA or (ii) NHE1 activity by using EIPA reduces proliferation/growth and induces apoptosis of HCC cells. EIPA also inhibits tumor growth in nude mouse xenografts of HCC cells, suggesting that inhibition of NHE1 could be a potential therapeutic target for the treatment of HCC (Yang et al., 2011). Di Sario et al. (2007) have also demonstrated that cariporide, a non-amiloride derivative, and other selective and potent inhibitors of NHE1 reduce proliferation and induce apoptosis in cholangiocarcinoma cells. Furthermore, in human PDAC cell lines, NHE1 drives both basal and EGF-stimulated three-dimensional growth and early invasion via invadopodial extracellular matrix digestion. Cariporide was found to reduce growth and invasion independently of the PDAC subtype and to sensitize these cells to low doses of erlotinib (Cardone et al., 2015), supporting the potential therapeutic value of cariporide.

### Targeting NHE3 Activity as a Treatment Modulator for Diarrhea and Irritable Bowel Syndrome

Secretory diarrhea usually results from abnormal fluid and electrolyte absorption and/or secretion. Electroneutral fluid absorption is carried out by the coordinated activity of NHE3 with Cl^–^/HCO_3_^–^ exchangers (Seidler, 2013; Xia et al., 2014). Studying cell cultures and rodent models revealed that NHE3 is inhibited in secretory diarrhea, and that NHE3 activity in enterocytes is inhibited under the conditions of acutely elevated intracellular concentrations of Ca^2+^, cAMP, or cGMP, caused by bacterial enterotoxins, humoral agonists or ionophores (Li et al., 2004; Gill et al., 2005). Enteroids generated from human proximal small intestine show an NHE3 activity which is stimulated by dexamethasone and inhibited upon exposure to rotavirus, cholera toxin and bacterial enterotoxins (Foulke-Abel et al., 2014). A peptide that mimics part of the NHE3 C-terminal domain prevents NHE3 inhibition by cAMP, Ca^2+^ and cholera toxin, suggesting the potential utility of NHE3 as a therapeutic target in such conditions.

Another study showed the use of tenapanor and its analogs in patients with constipation-predominant irritable bowel syndrome and for the treatment of hyperphosphatemia in end-stage renal disease patients on dialysis, implying that NHE3 inhibitors could be regarded as potential stool softeners in constipation-predominant irritable bowel syndrome. But both the safety and the long-term efficiency of NHE3 inhibition still remain unclear and need further study (Labonte et al., 2015; Zielinska et al., 2015).

## Conclusion and Perspective

There is a multitude of studies dealing with the physiology and pathophysiology of NHEs in the gastrointestinal tract. While it is indisputable that NHE1-3 and NHE8 play prominent roles in most if not even all segments of the gastrointestinal tract, the functions and the significance of NHE4, and NHA2 are rather inconclusive, so that further studies are needed indeed. Furthermore, observations made in, and the results obtained from, the various model systems such as cell culture, organoid, mouse and rat are hard to compare and cannot be directly applied to human beings. This specifically applies to the complex interaction between microbiota and intestinal epithelia, including the role of short chain fatty acids, which has gained more and more interest over the last two decades. There are studies showing that short chain fatty acids have an impact on the expression of NHE isoforms (Subramanya et al., 2007). Here the differences between the models are even more momentous because (i) cells in culture and organoids need to be mixed with short chain fatty acids and (ii) different species prefer different food leading to species-dependent differences in the composition of both microbiota and their short chain fatty acids. This flaw needs to be overcome in order to give more importance to the individual study with its results/observations. A first step could be a well-defined global standardization of the model systems and a systematic characterization of their differences and similarities based on which the findings and their impact could be assessed and classified.

## Author Contributions

LC conceived and wrote the manuscript. LC and ZY contributed to data acquisition, analysis, and interpretation. ML and CS critically revised the manuscript.

## Conflict of Interest

The authors declare that the research was conducted in the absence of any commercial or financial relationships that could be construed as a potential conflict of interest.

## Abbreviations

CD: Crohn’s disease
CRC: colorectal cancer
DMA: dimethylamiloride
DMH: 1,2-dimethylhydrazine dihydrochloride
DSS: dextran sulfate sodium
EGF: epidermal growth factor
EIPA: 5-(*N*-ethyl-*N*-isopropyl)-amiloride
EPEC: enteropathogenic *Escherichia coli;*
ESCC: esophageal squamous cell carcinoma
GERD: gastroesophageal reflux disease
HCC: hepatocellular carcinoma
HFD: high-fat-diet
HSC: hepatic stellate cells
IBD: inflammatory bowel disease
IFN γ: interferon γ
IL-1 β: interleukin 1 β
IL-4: interleukin 4
IL-6: interleukin 6
MMPs: matrix metalloproteinases
NAFLD: non-alcoholic fatty liver disease
NF-κB: nuclear factor “kappa-light-chain-enhancer” of activated B-cells
PDAC: pancreatic ductal adenocarcinoma
PDZK1: human gene encoding the Na^+^/H^+^ exchange regulatory co-factor NHE-RF3
PKA: protein kinase A
PKC: protein kinase C
TNBS: 2,4,6-trinitrobenzenesulfonic acid
TNF- α: tumor necrosis factor α
UC: ulcerative colitis
VEGF: vascular endothelial growth factor.
